# Hybrid procedure for patient with aortoectasia and aortic coarctation – case report

**DOI:** 10.1186/1749-8090-8-S1-O68

**Published:** 2013-09-11

**Authors:** N Hristov, T Anguseva, Z Mitrev

**Affiliations:** 1 Special Hospital for Surgery Fillip II, Skopje, Macedonia

## Background

Aortoectasia with a severe aortic regurgitation and coarctation of the descending aorta can be successfully treated with a hybrid strategy. Balloon expandable stents have been used to manage coarctation of the aorta, and in a second step Tyrone David reconstruction have been performed for reconstruction of the ascending aorta in to the normal morphology.

## Method

22y old patient with a history of a hypertensive disease, and frequent chest pain and fatigue had been diagnosed for aortoectasia (7cm ascending aorta) with a severe aortic regurgitation and aortic coarctation by echocardiography and multislice computered tomography.

## Result

In a first step patient got primary stenting with an immediate relief of the gradient. All antihypertensive medications were discontinued immediately. After 5 months patient got a surgery in a second step, preserving a nature aortic leaflets into the Dacron graft and with reimplantation of the both coronary arteries. Control transoesophageal echocardiography and CT scan showed normal morphology of the ascending aorta, no regurgitant jet trough the aortic valvula, no pressure gradient on the descending aorta.

## Conclusion

In patients with coarctation of the aorta and aortoectasia, stent implantation may be a feasible and improved option to relieve the stenosis in a first step, allowing for surgical reconstruction of the aortic root. Patient had a normal quality of life after surgery, follow up period 3.5 years.

